# No evidence for the presence of Epstein-Barr virus in squamous cell carcinoma of the mobile tongue

**DOI:** 10.1371/journal.pone.0184201

**Published:** 2017-09-19

**Authors:** Torben Wilms, Gulfaraz Khan, Philip J. Coates, Nicola Sgaramella, Robin Fåhraeus, Asma Hassani, Pretty S. Philip, Lena Norberg Spaak, Luigi Califano, Giuseppe Colella, Katarina Olofsson, Christos Loizou, Renato Franco, Karin Nylander

**Affiliations:** 1 Department of Clinical Sciences/ENT, Umeå University, Umeå, Sweden; 2 United Arab Emirates University, College of Medicine & Health Sciences, Dept. of Medical Microbiology and Immunology, Tawam Hospital Campus, Al Ain, UAE; 3 RECAMO, Masaryk Memorial Cancer Institute, Zluty kopec 7, Brno, Czech Republic; 4 Department of Medical Biosciences, Umeå University, Umeå, Sweden; 5 University Paris Diderot, INSERM UMRS1162, 27 rue Juliette Dodu, Paris, France; 6 Department of Neuroscience Reproductive and Dentistry Sciences, University of Naples Federico II, Naples, Italy; 7 Second University of Naples, Multidisciplinary Department of Medical, Surgical and Dental Specialties, Naples, Italy; 8 Dipartimento Universitario di Anatomia Patologica, Seconda Universita' Degli Studi di Napoli, Piazza Miraglia, Naples, Italy; University of North Carolina at Chapel Hill, UNITED STATES

## Abstract

Squamous cell carcinoma of the head and neck (SCCHN) comprises a large group of cancers in the oral cavity and nasopharyngeal area that typically arise in older males in association with alcohol/tobacco usage. Within the oral cavity, the mobile tongue is the most common site for tumour development. The incidence of tongue squamous cell carcinoma (TSCC) is increasing in younger people, which has been suggested to associate with a viral aetiology. Two common human oncogenic viruses, human papilloma virus (HPV) and Epstein-Barr virus (EBV) are known causes of certain types of SCCHN, namely the oropharynx and nasopharynx, respectively. EBV infects most adults worldwide through oral transmission and establishes a latent infection, with sporadic productive viral replication and release of virus in the oral cavity throughout life. In view of the prevalence of EBV in the oral cavity and recent data indicating that it infects tongue epithelial cells and establishes latency, we examined 98 cases of primary squamous cell carcinoma of the mobile tongue and 15 cases of tonsillar squamous cell carcinoma for the presence of EBV-encoded RNAs (EBERs), EBV DNA and an EBV-encoded protein, EBNA-1. A commercially available *in situ* hybridisation kit targeting EBER transcripts (EBER-ISH) showed a positive signal in the cytoplasm and/or nuclei of tumour cells in 43% of TSCCs. However, application of control probes and RNase A digestion using in-house developed EBER-ISH showed identical EBER staining patterns, indicating non-specific signals. PCR analysis of the BamH1 W repeat sequences did not identify EBV genomes in tumour samples. Immunohistochemistry for EBNA-1 was also negative. These data exclude EBV as a potential player in TSCC in both old and young patients and highlight the importance of appropriate controls for EBER-ISH in investigating EBV in human diseases.

## Introduction

Squamous cell carcinoma of the head and neck (SCCHN) comprises a large group of tumours in the head and neck region and occurs mainly in older people who have a history of alcohol and tobacco use. Viruses have also been implicated in the pathogenesis of SCCHN at specific sites, including Epstein-Barr virus (EBV) in undifferentiated nasopharyngeal cancer (uNPC) [1, 2] and human papilloma virus (HPV) in oropharyngeal squamous cell carcinomas (SCC) [3].

Within the oral cavity, the mobile tongue is the most common site for SCC and a viral aetiology has been suggested as one possible cause of the increasing incidence of tongue SCC (TSCC) in young non-smokers and non-drinkers [4–6]. We have recently shown that TSCCs are not associated with HPV [7], leaving EBV as a potentially causative oncogenic virus in this tumour type. The majority of adults in the world have been infected with EBV and the virus establishes a persistent latent infection [8]. Primary infection generally occurs early in life through oral transmission from parent to child and is usually asymptomatic. During the natural life cycle of EBV, persistent latent infection occurs in B-lymphocytes and epithelial cells in the nasopharynx are thought to be the site for viral replication and shedding into the saliva [8, 9]. This general picture may not be entirely accurate, with recent evidence that EBV may also establish latency in basal epithelial cells in the oral cavity (reviewed in [10]).

Although the majority of infected individuals never develop a viral-related disease, EBV has oncogenic properties and is aetiologically associated with several human malignancies, including various lymphomas, gastric cancer and uNPC, where EBV exists in a latent state [1, 9, 11]. Within the oral cavity, EBV is most well known in association with oral hairy leukoplakia (OHL), a lesion found mainly on the lateral tongue in severely immunocompromised patients. In this condition, virus is shed from the apical epithelium with lytic viral replication occurring in the differentiated cells of the upper epithelial layer [12, 13]. More recently, evidence for latent virus in the undifferentiated and longer living basal cells in OHL and the normal tonsil has been reported [14], suggesting that EBV may persist in epithelial cells of the oral cavity. In contrast to uNPC, there is limited and inconsistent evidence for an involvement of EBV in the pathogenesis of oral SCCs. Some studies have indicated that EBV is prevalent [15–20], whilst others have reported that it is relatively rare [21], or commonly seen but unlikely to have an aetiological role [22, 23]. To some extent, these inconsistencies could be attributed to the different methodologies used. Highly sensitive detection methods may not be appropriate for identifying a causal relationship, especially for viruses such EBV that are highly prevalent in the population [22, 23]. Moreover, aetiologically associating EBV with a given malignancy requires an *in situ* approach to avoid false implications. For example, in Hodgkin’s disease EBV is regularly seen in the non-malignant lymphocytic component [24]. This can lead to overestimation of EBV-associated disease using techniques such as PCR [24]. Additionally, some studies have reported high levels of viral DNA, but inconsistent expression of viral mRNA or protein in malignant cells [20, 22]. Others find cytoplasmic/nuclear rather than membrane expression of LMP1 [17], casting doubt on the validity of the findings for an aetiological role.

Latently EBV infected cells express up to six nuclear antigens, of which EBNA-1 is critical for maintaining the viral genome in infected cells and is a potential target for therapies against EBV-associated diseases [25]. There are also two non-coding RNAs, EBER1 and EBER2, which are the most abundant viral transcripts in infected cells. Functionally, EBERs are thought to inhibit apoptosis and induce cell proliferation [26, 27]. Due to their abundance and presence in all EBV infected cells, detection of EBERs using *in situ* hybridization is commonly used in clinical practice and research to identify the presence of the virus [26].

Given that: 1) the incidence of TSCC is increasing in young patients without exposure to the classical carcinogenic agents associated with this disease, 2) EBV infection is prevalent in the oral cavity, and 3) EBV is now recognised to infect and establish latency in oral epithelium, including the tongue, we have re-investigated the potential role for EBV as a causative agent in TSCC.

## Materials and methods

### Tissue samples

Ninety eight cases of primary squamous cell carcinoma of the mobile tongue (TSCC) and 15 tonsillar SCC were included in the analysis. Of the TSCCs, 86 were retrieved from Clinical Pathology at the University Hospital of Umeå, NUS, and 12 cases from the Second University of Naples, Multidisciplinary Department of Medical, Surgical and Dental Specialties, Naples, Italy. The 15 cases of tonsillar cancer were all from Clinical Pathology at the University Hospital of Umeå. Fifty of the tongue carcinomas and all of the tonsillar carcinomas had previously been analysed for the presence of HPV [7, 28].

Patients were diagnosed at ages ranging from 19–93 years, with a mean age of 63.7 years; 49% were over 65 years, 39% between 41–65 years and the remaining 12% ≤ 40 years. None of the patients with tonsillar SCC were in the group of young patients. Thirty tumours were T1, 43 were T2 and of the remaining 40, half were T3 and the other half T4. The majority of tumours (77) were N0. The study was granted by the Regional Ethics Review Board, Umeå, Sweden (dnr 03–201). As archived tissue was used no written consent was required.

### EBER in situ hybridisation

EBER-*in situ* hybridisation (EBER-ISH) was performed on all 98 TSCCs and the 15 tonsillar SCCs using a commercially available kit. Briefly, two sections were cut from each tumour and in one, the Inform EBER Probe was used for detection of EBER1 and EBER2 (800–2842, Ventana Medical Systems, Roche Diagnostics GmbH, Mannheim Germany). In parallel, the other section was incubated with RNA Positive Control Probe (800–2846, Ventana) to assure presence of mRNA in the sample. NBT-BCIP Detection Chemistry was used for visualisation (800–092, Roche Diagnostics GmbH, Mannheim, Germany). The staining procedures were performed in a Bench Mark Ultra (Ventana Medical Systems, Inc, Tucson, AZ, USA) according to guidelines from the supplier.

For independent verification of the results obtained using the commercial EBER-ISH kit, 10 TSCCs were re-tested using a non-commercial protocol with custom synthesised oligonucleotides and in-house labelling as previously described [29, 30]. Briefly, tissue sections were deparaffinised, endogenous tissue peroxidase activity blocked by incubating in 0.5% H_2_O_2_ in methanol for 20 minutes and tissues digested with 100μg/ml proteinase K for 10 min at 37°C. Sections were subsequently washed in water, dehydrated and hybridised overnight at 42°C using a mixture of digoxigenin-labeled EBER-1 and EBER-2 anti-sense oligonucleotide probes complementary to sequences 91–120 of EBER-1 and 82–111 of EBER-2. After stringency washes in 0.1x SSC at 50°C, hybridised probes were detected using mouse anti-digoxin monoclonal antibody (clone D1-22, Sigma D8156) and Avidin-Biotin-Complex peroxidase reagents (ThermoScientific 32052) using DAB as chromogen. Sections were counterstained with haematoxylin, mounted in a permanent mount and examined by light microscopy. Sections from a case of EBV-positive infectious mononucleosis were used as a positive control (kind gift of Prof Gerald Niedobitek, Berlin). Those cases that gave a positive signal were subjected to two control procedures to assess signal specificity. One tissue section from each case was pre-treated with RNase A prior to hybridisation with anti-sense probes. In addition, one section was processed using digoxigenin-labelled EBER-1 and EBER-2 sense probes.

### PCR for EBV

From nine of the ten TSCC cases mentioned above, five 10 μm sections were cut from the paraffin blocks. DNA was extracted using the QIAamp® DNA Mini Kit (Qiagen) and diluted in 200μl nuclease-free water. The total amount of DNA extracted varied between 0.84–18.22μg. PCR was performed on 100ng of DNA using primers specific for EBV BamHI ‘W’ fragment [31]. Amplification of *HBB* (human β-globin gene) was performed to assess DNA integrity [32]. Each PCR reaction contained 0.4μM primers and 1 unit of QiaGen Taq DNA polymerase in a total volume of 30μl and amplified for 30 cycles. For each PCR run, a positive control (DNA from EBV-positive cell line B95-8) and a negative control (nuclease-free water) was included. Amplified products were visualised on ethidium bromide stained 2% agarose gels. The expected size of the amplicon was 152bp for EBV BamH1W and 104bp for *HBB*.

### Immunohistochemistry for EBNA-1

Tissue sections from tumour samples were dewaxed and rehydrated. Antigen retrieval was achieved by boiling the slides in Tris buffer (pH 9) for 20 minutes and endogenous peroxidase activity was blocked in 0.5% H_2_O_2_. Sections were blocked in 5% BSA at room temperature for 2hrs and incubated with mouse anti-EBNA-1 antibody (clone D810H, ThermoFisher) at a 1:50 dilution. Signals were detected using the ABC peroxidase staining kit (Thermo Scientific, Cat# 32052) and DAB as chromogen. Sections were counterstained with haematoxylin, dehydrated and mounted for microscopic analysis. Formalin-fixed paraffin embedded sections from an EBV infected rabbit spleen [33] were included as a positive control.

## Results

**Fig 1:**
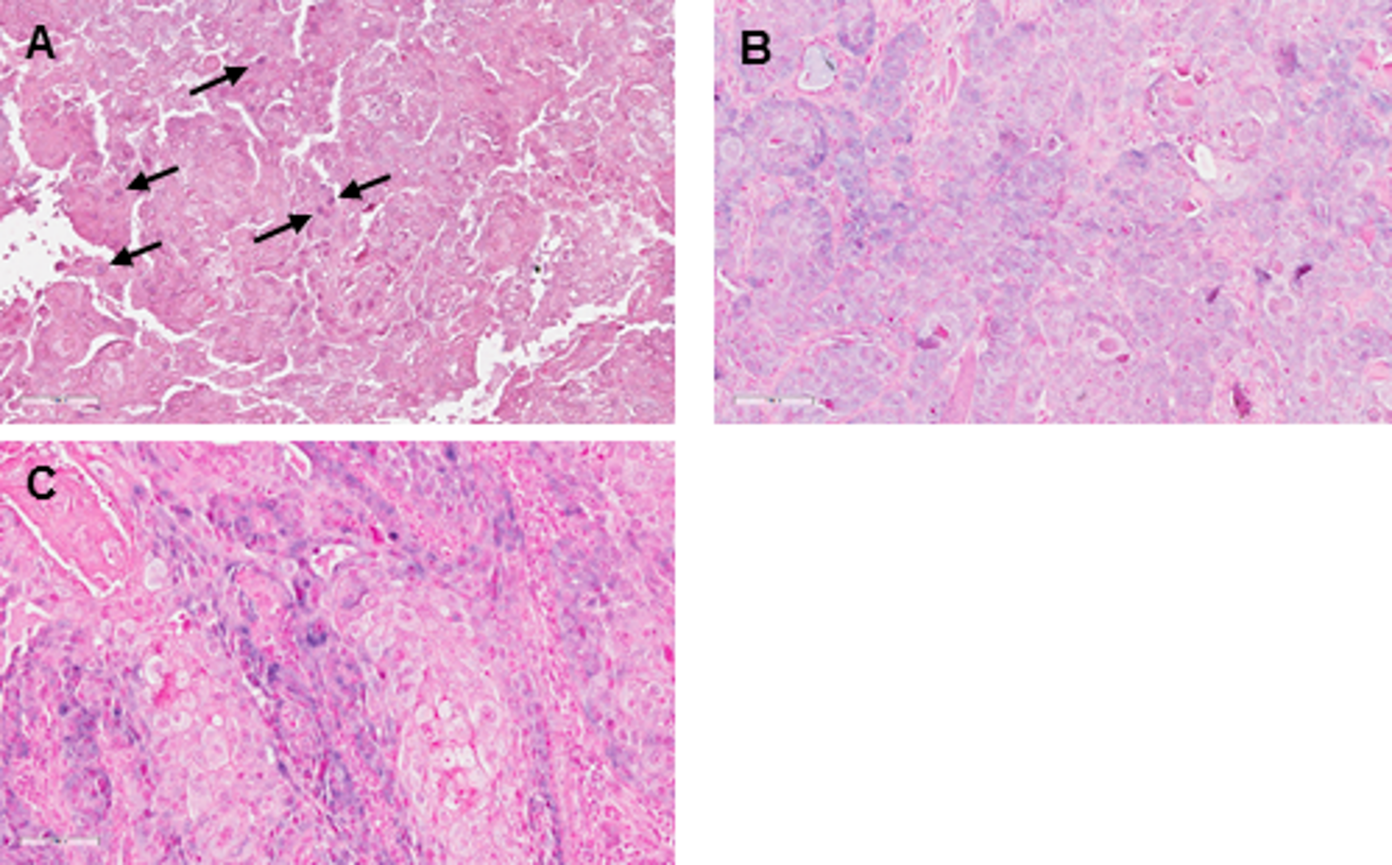
EBER *in situ* hybridization (EBER-ISH) using commercially labelled probes and detection reagents. Signals are detected as a purple colour and nuclei are countrerstained red. A) shows an example of TSCC where most cells are negative and a few tumour cells show faint nuclear staining (arrows). In tumour B) there is a predominantly cytoplasmic staining pattern in the majority of tumour cells, with some nuclear staining also seen. C) An example of a TSCC showing strong signals in the cytoplasm of tumour cells, but without nuclear staining.

EBER-ISH using the commercial kit was performed in the Clinical Pathology laboratory in Umea on all 113 carcinomas. All samples showed staining with the positive control probe, indicating suitability of these tissues for the method. Of the 98 TSCCs, 56 (57%) were negative, 41 cases (42%) showed cytoplasmic positivity and 5 of these also showed occasional cells with nuclear positivity. One case (1%) showed nuclear positivity only. For the 15 tonsillar SCC, 7 (46.5%) were negative, one case (7%) showed occasional positive nuclei and 7 cases (46.5%) showed cytoplasmic positivity (Fig 1). In all of the samples that gave positive EBER signals, the staining was not seen in all tumour cells. Moreover, the staining was not strong, discrete and nuclear with nucleolar sparing, as is typically seen for EBER-ISH in other studies [24, 30].

**Fig 2:**
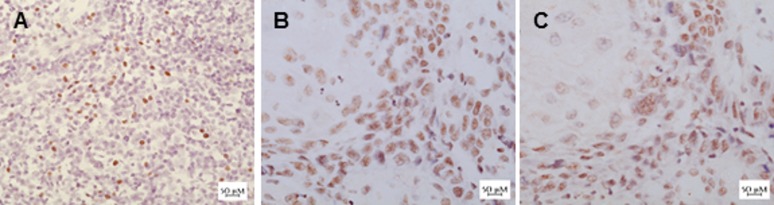
EBER *in situ* hybridization (EBER-ISH) using in-house prepared antisense and sense probes. Signals are detected with DAB (brown) and nuclei are counterstained with haematoxylin (blue). A) Strong nuclear signal characteristic of EBER-ISH staining in tonsil from a patient with infectious mononucleosis used as a positive control. B) EBER-ISH of TSCC showing diffuse and speckled nuclear staining. C) Similar signals were seen using non-complementary sense probes.

Of the TSCC cases with nuclear and/or cytoplasmic EBER reactivity, 10 were chosen for further analysis using in-house prepared probes and reagents for EBER-ISH. The application of digoxigenin-labelled anti-sense EBER1 and EBER2 oligonucleotides showed a similar pattern of staining in each sample (Fig 2). Remarkably, the same pattern of staining was seen after RNase A treatment prior to hybridisation with anti-sense probe. Moreover, similar signals were also seen when the anti-sense probes were replaced with digoxigenin-labelled non-complementary sense probes (Fig 2). Furthermore, as with the commercial EBER ISH kit, EBER staining using the in-house protocol also gave weak and diffuse patterns. By contrast strong nuclear staining with nucleolar sparing was seen in the EBV positive control sections (Fig 2).

**Fig 3:**
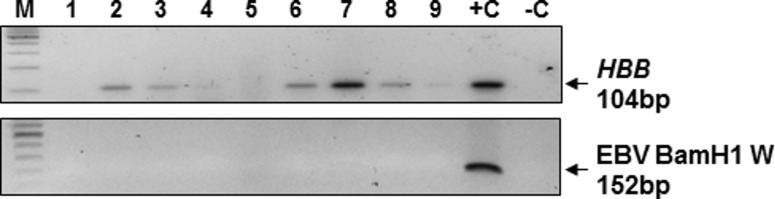
PCR for *HBB* and EBV BamH1 W fragment. A 30 cycle PCR on 100 ng of gDNA showing amplification of *HBB* at 104 bp. *HBB* could not be amplified in samples 1 and 5. No amplification of EBV BamH1 W was seen in any sample. M = 100bp DNA marker; +C = positive control (100ng DNA extracted from the EBV-positive B95-8 cell line); -C = negative control (nuclease-free water).

As an independent test for EBV in these samples, DNA was extracted and PCR performed for EBV DNA. Of the nine cases with sufficient material available for study by this method, *HBB* could be amplified in seven, indicating that the DNA was of suitable quality and quantity for PCR amplification (Fig 3). None of the TSCC cases were positive for EBV using primers targeting the BamH1 ‘W’ region. EBV BamHI W region is present in multiple copies (7–12 copies) within the viral genome [34]), making PCR amplification of this region a highly sensitive method for the detection of EBV.

**Fig 4:**
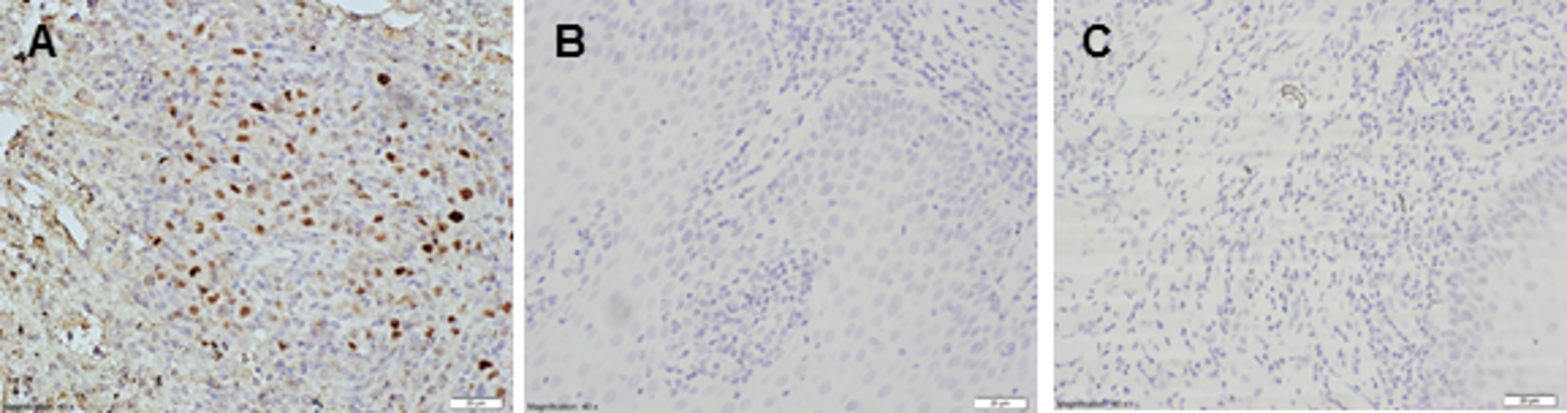
Immunostaining for EBV EBNA1. The presence of EBNA1 is detected with DAB (brown) and nuclei are counterstained with haematoxylin (blue). A) Positive control (EBV infected rabbit spleen) shows widespread nuclear staining. B) and C) show images of two representative TSCCs.

As a further independent test for EBV, immunohistochemistry for EBNA1 was performed on the 10 samples. Strong nuclear staining for EBNA1 was evident in the EBV-infected rabbit spleen used as positive control. In contrast, none of the TSCCs showed EBNA1 staining (Fig 4).

## Discussion

Viruses are attracting much attention as potential aetiological factors for TSCC (see [35–38] for recent reviews). We have previously used PCR for all HPV types and *in situ* hybridisation for HPV16, the most common high risk HPV seen in association with SCCHN. We were not able to detect HPV in any of the TSCC cases studied [7]. In contrast, tonsillar SCC showed HPV infection in 91% of the cases [28], in keeping with the known role of HPV in SCCs arising in the oropharyngeal region [3]. As HPV is not the only oncogenic virus present in the oral cavity and implicated in the development of TSCC, we investigated whether there is evidence for a potential role of EBV in this tumour type. The potential for EBV as a causative factor for TSCC is particularly relevant in view of its prevalence in the oral cavity and its known ability to infect tongue epithelial cells [10]. Moreover, a viral cause is a plausible explanation to account for the increasing incidence of TSCC in young patients who have not been exposed to the classical aetiological factors for this disease [4–6]. In addition, many of the previous studies were performed before the increased incidence of TSCC in young patients became apparent, so that this particular group would not have been represented in those studies.

Using a commercially available kit for detection of two non-coding EBV RNAs, EBER1 and EBER2, 42 of the 98 TSCCs (43%) analysed showed cytoplasmic and/or nuclear positivity. The staining pattern for EBERs was not typical of that reported in EBV associated malignancies; strong nuclear staining with nucleoli sparing [24, 30, 39]. Indeed, when trying to verify these results using a well-established in-house protocol, signals were seen also in the negative control sections pre-treated with RNase A to digest the targets prior to hybridisation. Similar staining patterns were seen also in a second negative control using non-complementary sense EBER probes, indicating that the hybridisation signals were non-specific. Further investigation using PCR was unable to identify viral DNA sequences in these tumour samples. Our PCR method amplifies the BamH1 W fragment of EBV, which is a sequence with multiple repeats in the viral genome and is therefore a highly sensitive method and able to detect low viral copy numbers. Indeed, in samples in which an endogenous gene can be amplified under the same conditions, an average of 10 BamH1 W repeats per EBV genome [34] implies that a single viral copy/cell can be detected, even when the tumour represents only 20% of the sample. Thus, the use of higher numbers of amplification cycles is both unnecessary and inappropriate, because very high sensitivity will lead to viral detection that is not associated with the tumour, as has been noted previously [15, 24]. Finally, an independent method to assess EBV activity by immunohistochemistry did not demonstrate expression of EBNA1 protein in any TSCC. Since EBNA1 is required for viral maintenance, a lack of staining confirms the absence of viral activity in these tumours.

Taken together, our results do not support an association of EBV with TSCC in either young or old patients. The observation that these tumours also do not harbour HPV [7] indicates that neither of these oncogenic viruses account for the increasing incidence of this disease in younger people seen in the West [4–6]. Whilst our data and conclusions on EBV are in agreement with some reports [22], they contrast to other published data in which EBV has been associated with SCCHN [15–17, 21]. In many of these studies, orthogonal methods have either not been used to confirm viral presence [17] or have produced conflicting results [21]. In addition, many studies have used EBER-ISH as the primary method, which we have shown here to produce spurious staining results in some samples. Other than these technical considerations, the exact anatomical location from where the samples are collected is often not stated clearly. Hence it is difficult to judge whether the tumours tested were from the mobile tongue, as in our study, or from the base of tongue. Another factor to take into consideration is that many studies reporting an association of EBV with oral SCC originate in Asia [15, 16, 21, 40–42], the geographical area in which EBV-associated uNPC and gastric cancers are particularly prevalent [1, 9]. Thus, there may be an association between EBV and TSCC in other geographic locations due to differences in lifestyle, diet or ethnicity.

In summary we have used three different methods for the detection of EBV and its most prevalent products, the EBERs and EBNA-1, without finding evidence for presence of the virus in TSCC or tonsillar SCC. Combining the present data with our previous findings showing lack of HPV infection in TSCC [7] indicates that these two viruses are unlikely to be involved in the pathogenesis of squamous cell carcinomas of the mobile tongue. We were also unable to find evidence for dual infection of EBV with HPV in our oral SCCs, suggesting that the possible role of co-infection of these two viruses [35, 43] is not a prominent factor in SCCHN, at least at these two sites in a European population.
